# Involvement of NMDA receptors containing the GluN2C subunit in the psychotomimetic and antidepressant-like effects of ketamine

**DOI:** 10.1038/s41398-020-01110-y

**Published:** 2020-12-10

**Authors:** Mireia Tarrés-Gatius, Lluís Miquel-Rio, Leticia Campa, Francesc Artigas, Anna Castañé

**Affiliations:** 1 Institut d’Investigacions Biomèdiques de Barcelona (IIBB-CSIC), Barcelona, Spain; 2 Institut d’Investigacions Biomèdiques August Pi i Sunyer (IDIBAPS), Barcelona, Spain; 3 Centro de Investigación Biomédica en Red de Salud Mental (CIBERSAM), ISCIII, Madrid, Spain; 4 Universitat de Barcelona, Barcelona, Spain; 5 Facultat de Medicina, Universitat de Vic-Universitat Central de Catalunya (UVic-UCC), Vic, Spain

**Keywords:** Depression, Schizophrenia, Pharmacology

## Abstract

Acute ketamine administration evokes rapid and sustained antidepressant effects in treatment-resistant patients. However, ketamine also produces transient perceptual disturbances similarly to those evoked by other non-competitive NMDA-R antagonists like phencyclidine (PCP). Although the brain networks involved in both ketamine actions are not fully understood, PCP and ketamine activate thalamo-cortical networks after NMDA-R blockade in GABAergic neurons of the reticular thalamic nucleus (RtN). Given the involvement of thalamo-cortical networks in processing sensory information, these networks may underlie psychotomimetic action. Since the GluN2C subunit is densely expressed in the thalamus, including the RtN, we examined the dependence of psychotomimetic and antidepressant-like actions of ketamine on the presence of GluN2C subunits, using wild-type and GluN2C knockout (GluN2CKO) mice. Likewise, since few studies have investigated ketamine’s effects in females, we used mice of both sexes. GluN2C deletion dramatically reduced stereotyped (circling) behavior induced by ketamine in male and female mice, while the antidepressant-like effect was fully preserved in both genotypes and sexes. Despite ketamine appeared to induce similar effects in both sexes, some neurobiological differences were observed between male and female mice regarding *c-fos* expression in thalamic nuclei and cerebellum, and glutamate surge in prefrontal cortex. In conclusion, the GluN2C subunit may discriminate between antidepressant-like and psychotomimetic actions of ketamine. Further, the abundant presence of GluN2C subunits in the cerebellum and the improved motor coordination of GluN2CKO mice after ketamine treatment suggest the involvement of cerebellar NMDA-Rs in some behavioral actions of ketamine.

## Introduction

Major depressive disorder (MDD) is predicted to be the second leading cause of illness-induced disability by 2030^1^, causing a very large socioeconomic burden^2,3^. The World Health Organization^4^ estimates to affect 322 million people, a 4.4% worldwide point prevalence, being more common among females (5.1%) than males (3.6%). This high prevalence, together with the slow onset of clinical action and limited efficacy of standard monoaminergic treatments^5^, make it necessary to develop better therapeutic approaches, especially for those patients with suicidal ideation. In this context, a single administration of the non-competitive N-methyl-d-aspartate receptor (NMDA-R) antagonist ketamine evokes fast (in hours) and persistent (up to 1 week) antidepressant responses in patients with treatment-resistant depression (TRD)^6–8^. Recently, intranasal esketamine (the S-isomer of ketamine) was approved by the US Food and Drug Administration for TRD when used in conjunction with conventional oral antidepressants^9,10^. However, ketamine evokes side effects such as sedation, as well as transient perceptual disturbances that resemble those of a psychotic-dissociative-state^11,12^. Even though these side effects are more pronounced in men than in women^13^, to date, there is no evidence of gender being a significant predictor of ketamine’s antidepressant efficacy^14–16^. Moreover, the high risk of abuse potential with ketamine may limit its long-term use^17^. Hence, there is an urgent need to understand the basis of the psychotomimetic and antidepressant effects of ketamine in order to develop new fast-acting antidepressants devoid of pro-psychotic actions and abuse potential.

Although the exact mechanism of non-competitive NMDA-R antagonists-including ketamine- to produce antidepressant and psychotomimetic effects is not fully understood^18^, the most prevalent view is that they may preferentially target NMDA-Rs in GABAergic interneurons^18–20^ in order to increase cortical pyramidal neurons activity. Likewise, previous studies by our group have shown that phencyclidine (PCP) and ketamine also block NMDA-Rs in GABAergic neurons of the reticular thalamic nucleus (RtN), which tonically inhibits the rest of excitatory thalamic nuclei^21,22^. In the case of PCP, this effect translates into an overall disinhibition of the excitatory thalamic nuclei and a marked activation of thalamo-cortical networks in the anesthetized rats^23–25^. Ketamine, despite inhibiting RtN neurons, evokes a further inhibition of thalamo-cortical activity in anesthetized rats^21^. However, both agents show a similar action in freely moving rats, enhancing the activity of thalamic and cortical neurons, including that of GABAergic interneurons^26^, which suggests a mechanism of action different from that of MK-801^19^, at least in cortical networks.

Since thalamo-cortical networks are involved in the processing of sensory information, among others, their activation by PCP and ketamine may underlie psychotomimetic actions. This view is also supported by the normalization of thalamo-cortical activity after PCP by antipsychotic drugs with different mechanism of action^24,25,27,28^.

The action of ketamine may not be limited to NMDA-R blockade. Preclinical studies pointed to an activation of α-amino-3-hydroxy-5-methyl-4-isoxazolepropionic acid receptors (AMPA-Rs) by its metabolite hydroxyl-norketamine (HNK)^29^, increased mTOR signaling and BDNF-dependent synaptogenesis^30,31^. However, HNK preclinical findings seem not translate to human clinical depression, as discussed recently^32^, and should be further investigated.

It has been suggested that ketamine may preferentially act on GluN2C and/or GluN2D-containing NMDA-Rs rather than on GluN2B and GluN2A-containing receptors^33^ to produce psychosis^34^. GluN2C-containing NMDA-Rs are highly expressed in olfactory bulbs, cerebellum^35–38^, as well as in thalamic nuclei, including the RtN^39–41^. During brain development, the GluN2C subunit contributes to synaptic currents in the cerebellum^42,43^. Given the involvement of thalamo-cortical networks in the actions of NMDA-R antagonists, we examined the dependence of psychotomimetic and antidepressant-like actions of ketamine on the presence of this subunit, under the working hypothesis that psychotomimetic -but less probably the antidepressant-like effects- would be attenuated in mice lacking the GluN2C subunit. Moreover, since MDD is more prevalent in women^44^, and given the scarcity of ketamine studies in female rodents, we conducted the present study in male and female mice.

## Materials and methods

### Subjects

Male and female WT and GluN2CKO mice backcrossed onto a C57BL/6J genetic background (achieving > 99% homogeneity) were used. The GluN2CKO colony was established in our animal facility after the generous donation of mice by Dr. Andrés Buonanno (National Institute of Child Health and Human Development, Bethesda, MD, USA) and Dr. Maria Cecilia Scorza (Instituto de Investigaciones Biológicas Clemente Estable, Montevideo, Uruguay). Tests were conducted at 8–12 weeks-of-age and in alternate weeks in male and female mice. Different sets of animals were used in the different tests. Animals were maintained in the animal facilities of the School of Medicine of the University of Barcelona, in a controlled environment (12-h light/dark cycle, 22 ± 1 °C room temperature) with ad libitum access to food and water. All experimental procedures were conducted in accordance with national (Royal Decree 53/2013) and European legislation (Directive 2010/63/EU, on the protection of animals used for scientific purposes, 22 September 2010), and were approved by the Institutional Animal Care and Use Committee of the University of Barcelona.

### Drugs

Ketamine (Ketolar^®^, Pfizer; 10–30 mg/kg) was dissolved in saline and injected intraperitoneally (i.p.). Doses of ketamine were chosen according to literature^45^ and are expressed as free base. The volume of injection was 4 ml/kg. All other drugs and reagents used were of analytical grade.

### Behavioral studies

#### Open field (OF)

Mice were placed in a dimly lighted (20–30 lx) arena (35 × 35 cm) during a 30-min trial immediately after ketamine/saline administration. Total distance moved, as well as distance moved in the periphery and center of the OF, was analyzed using the SMART 3.0 video tracking software (Panlab/Harvard apparatus, Cornellà del Llobregat, Spain). In addition, the number of rearings and falls (during horizontal displacement or by loss of balance while performing rearings), as well as the intensity of hindlimb abduction and circling were scored using a graded scale (0, absent; 1, equivocal; 2, present; and 3, intense) by a trained experimenter blind to mice genotype as previously described^46,47^. Mice were naive to the OF and were used only once. The OF was cleaned with 10% ethanol between subjects.

#### Tail suspension test (TST)

Mice were tested 30 min after ketamine/saline administration. They were suspended 30 cm above the floor by adhesive tape placed ~1 cm from the tip of the tail. Sessions were videotaped for 6 min and the immobility time was determined by a trained experimenter blind to mice genotype and treatment.

### Intracerebral microdialysis studies

Extracellular serotonin (5-HT) and glutamate (Glu) concentrations were measured by in vivo intracerebral microdialysis in freely moving mice as previously described^48,49^. Briefly, concentric dialysis probes (2 mm membrane length) were implanted unilaterally in the medial prefrontal cortex (mPFC) of anaesthetized mice (coordinates in mm: AP: +2.2; ML: −0.2; DV: −3.4 taken from bregma and top of skull). Experiments were performed 24–48 h after surgery. Probes were continuously perfused with artificial cerebrospinal fluid (aCSF) at a rate of 1.65 μl/min. The aCSF contained 1 μM citalopram in order to locally block 5-HT reuptake and estimate ketamine effects on 5-HT release (extracellular neurotransmitter concentrations reflect the physiological balance between release and reuptake). Thirty-min samples were collected after a stabilization period of 3 h. Neurotransmitter concentrations were determined by high performance liquid chromatography (HPLC) with electrochemical (5-HT) or fluorimetric (Glu) detection by a trained experimenter blind to mice genotype and treatment. Baseline neurotransmitter levels were calculated as the average of the four pre-drug samples. At the end of the experiments, mice were euthanized, and brains were removed to proceed with the histological verification of probes placement. Only data obtained from animals with histologically correct probe placements were used for subsequent statistical analysis.

### In situ hybridization studies

Mice were euthanized 1 h after treatment and brains were rapidly removed, frozen on dry ice and stored at −30 °C until processed. Brain coronal sections (14 μm thick) were cut using a microtome-cryostat (HM500-OM, Microm, Germany), thaw-mounted onto APTS (3-aminopropyltriethoxysilane, Sigma, MO, USA)-coated slides, kept at −30 °C and fixed in 4% paraformaldehyde. The primer sequence of mRNA for *c-fos* (gene ID: NM_022197.1) was 5′-GACCATGATGTTCTCGGGTTTCAACGCGGACTACGAGGCGTCATCCTC -3′. The oligonucleotide was labeled with [^33^P]-dATP (42500 Ci mmol^−1^; DuPont-NEN, MA, USA) with terminal deoxynucleotidyltransferase (Calbiochem, CA, USA) and purified with ProbeQuant G-50 Micro Columns (GE Healthcare UK Limited, UK), as described previously^50^. Hybridized sections were exposed for 7 days to Biomax MR film (Kodak, Sigma-Aldrich, Spain) with intensifying screens. Relative mean grey values (MGV) (arbitrary units) were measured with an image analyzer (Fiji, Madison, WI, USA). Two consecutive brain sections at any level of interest (AP coordinates from atlas Franklin and Paxinos^51^) were analyzed for each mouse and averaged to obtain individual values.

### Resting state functional magnetic resonance imaging (fMRI) studies

Mice were scanned on a 7.0 T BioSpec 70/30 horizontal animal scanner (Bruker BioSpin, Ettlingen, Germany), equipped with an actively shielded gradient system (400 mT/m, 12 cm inner diameter). Animals were sedated (medetomidine or isoflurane) and placed in supine position in a Plexiglas holder. 3D-localizer scans were used to ensure accurate position of the head at the isocenter of the magnet. Resting state fMRI with an echo planar imaging (EPI) sequence was acquired with the following parameters: 0.208 × 0.208 × 0.75 voxel size, TR/TE = 2000/19.4 ms, 420 repetitions and 14 min scan time before and after ketamine administration. After completion of the imaging session, atipamezol (Antisedan^®^, Pfizer) and saline were injected to reverse the sedative effect and compensate fluid loss. Resting-state fMRI was preprocessed including slice timing correction, motion correction by spatial realignment (using SPM toolbox (http://www.fil.ion.ucl.ac.uk/spm/software/spm12)), skull-strip based on Otsu thresholding^52^, detrending, spatial smoothing with a full-width half maximum (FWHM) of 0.6 mm and frequency filtering between 0.01 and 0.1 Hz. Six regions of interest (PFC, caudate-putamen nuclei (CPu), thalamus, motor cortex, cerebellum, and hippocampus (HPC)) were delineated in the left hemisphere on the mean volume and average time-series in each region was computed. Correlation between each pair of regional time-series was computed to quantify functional connectivity between regions.

### Statistical analysis

The number of animals used for each test is reported in the figure legends. Sample sizes were determined based on power analysis and common practice in behavioral (≈10 animals per group) and in situ hybridization studies (≈5 animals per group). No randomization method was used, and the experiments shown were only performed once in the laboratory.

Before the analysis, all data was checked for normality (Anderson–Darling; D’Agostino–Pearson omnibus; Shapiro–Wilk; Kolmogorov–Smirnov) and homogeneity of variances (Bartlett’s test and Brown–Forsythe test).

The data are expressed as mean ± SEM. Statistical analysis was carried out using unpaired *t*-test (with Welch’s correction when appropriate), two-way analysis of variance (ANOVA) and three-way ANOVA followed by Newman–Keuls post hoc comparisons after significant interactions between factors. When ANOVA assumptions were violated, Kruskal–Wallis test corrected for multiple comparisons using Dunn’s test was performed. As pre-established, outlier values (Chauvenet’s criterion) were excluded from all analyses. In all cases, the level of significance was set at *p* < 0.05.

## Results

### Behavioral syndrome

**Fig. 1:**
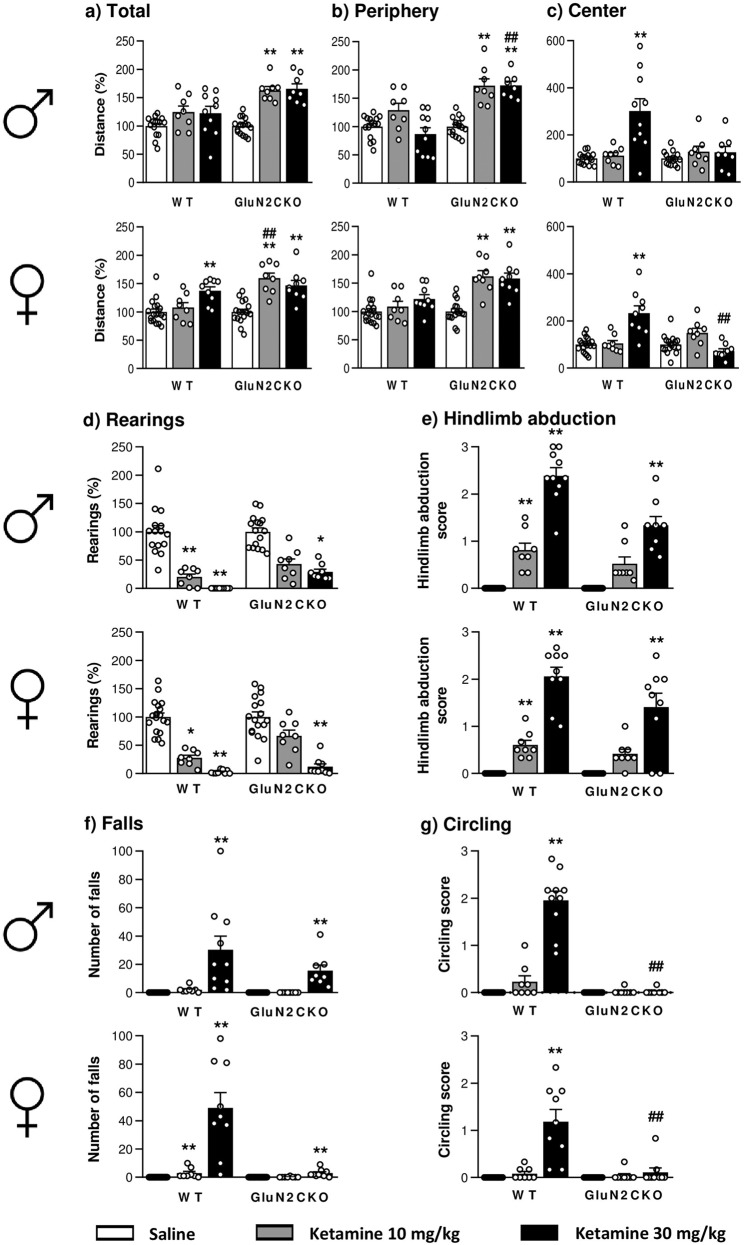
Behavioral syndrome induced by ketamine in WT and GluN2CKO mice of both genders. Effects of ketamine (10 and 30 mg/kg) in male (*♂*) and female (♀) WT and GluN2CKO mice on: **a** total distance (percentage from saline-treated mice); **b** peripheral distance (percentage from saline-treated mice); **c** central distance (percentage from saline-treated mice); **d** rearings (percentage from saline-treated mice); **e** hindlimb abduction; **f** falls and **g** circling. Male WT saline (*n* = 15); male GluN2CKO saline (*n* = 16); female WT saline (*n* = 18); female GluN2CKO saline (*n* = 17); male and female WT and GluN2CKO ketamine 10 mg/kg and male GluN2CKO ketamine 30 mg/kg (*n* = 8); male WT ketamine 30 mg/kg (*n* = 10); female WT and GluN2CKO ketamine 30 mg/kg (*n* = 9). *p < 0.05, **p < 0.01 vs. saline; ^##^*p* < 0.01 vs. WT (Dunn’s or Newman–Keuls post hoc tests).

Acute ketamine administration increased locomotor activity, especially in GluN2CKO mice (Fig. 1a). For male mice, there were significant group differences (Kruskal–Wallis test = 34.04; *p* < 0.0001) with a significant increase in total locomotor activity in ketamine-treated GluN2CKO mice compared to saline-treated mice (*p* < 0.01, all cases). For female mice, two-way ANOVA showed a main effect of treatment (*F*_2,63_ = 24.71; *p* < 0.0001), genotype (*F*_1,63_ = 12.40; *p* < 0.01) and treatment × genotype interaction (*F*_2,63_ = 7.32; *p* < 0.01). Post hoc comparisons revealed a significant increase of distance moved in ketamine-treated WT mice (ketamine 30 mg/kg, *p* < 0.01) and ketamine-treated GluN2CKO (*p* < 0.01 vs. saline, all cases). Moreover, a significant difference between WT and GluN2CKO mice treated with ketamine 10 mg/kg (*p* < 0.01) was also found.

Acute ketamine administration increased thigmotaxis, especially in GluN2CKO mice (Fig. 1b). For male mice, there were significant group differences (Kruskal–Wallis test = 36.82; *p* < 0.0001) with a significant increase in peripheral locomotor activity in ketamine-treated GluN2CKO mice compared to saline-treated mice (*p* < 0.01, all cases). Moreover, a significant difference between WT and GluN2CKO mice treated with ketamine 30 mg/kg (*p* < 0.01) was also found. For female mice, there were significant group differences (Kruskal–Wallis test = 33.53; *p* < 0.0001) with a significant increase in peripheral locomotor activity in ketamine-treated GluN2CKO mice compared to saline-treated mice (*p* < 0.01, all cases).

Ketamine (30 mg/kg) increased the distance moved in the center of the OF in male and female WT mice (Fig. 1c). For male mice, there were significant group differences (Kruskal–Wallis test = 17.11; *p* < 0.01) with a significant increase in locomotor activity in WT mice (*p* < 0.01). For female mice, there were significant group differences (Kruskal–Wallis test = 26.49; *p* < 0.0001) with a significant increase in locomotor activity in WT mice (*p* < 0.01) and a significant reduction was found in GluN2CKO, as compared with WT mice (*p* < 0.01).

Ketamine administration reduced the number of rearings (Fig. 1d). For male mice, there were significant group differences (Kruskal–Wallis test = 50.89; *p* < 0.0001) with a significant decrease in rearings in ketamine-treated WT mice (*p* < 0.01, all cases) and GluN2CKO mice (ketamine 30 mg/kg, *p* < 0.05). For female mice, there were significant group differences (Kruskal–Wallis test = 50.08; *p* < 0.0001) with a significant decrease in rearings after ketamine 30 mg/kg in WT and GluN2CKO mice (*p* < 0.01, all cases) and after ketamine 10 mg/kg in WT mice (*p* < 0.05).

Ketamine administration increased hindlimb abduction (Fig. 1e). There were significant group differences for male (Kruskal–Wallis test = 60.83; *p* < 0.0001) and female (Kruskal–Wallis test = 56.97; *p* < 0.0001) mice. In both sexes, ketamine increased hindlimb abduction in WT mice (*p* < 0.01, all cases) and ketamine 30 mg/kg increased hindlimb abduction in GluN2CKO mice (*p* < 0.01, all cases).

Ketamine (30 mg/kg) administration significantly increased the number of falls (Fig. 1f). There were significant group differences for male (Kruskal–Wallis test = 59.95; *p* < 0.0001) and female (Kruskal–Wallis test = 58.80; *p* < 0.0001) mice. In both sexes, ketamine 30 mg/kg increased the number of falls in both genotypes (*p* < 0.01, all cases). Moreover, for female mice, ketamine 10 mg/kg increased the number of falls in WT mice (*p* < 0.01).

Ketamine administration (30 mg/kg) increased circling behavior only in WT mice (Fig. 1g). There were significant group differences for male (Kruskal–Wallis test = 49.65; *p* < 0.0001) and female (Kruskal–Wallis test = 45.73; *p* < 0.0001) mice. In both sexes, there was a significant increase in circling behavior in ketamine-treated vs. saline-treated WT mice and a significant difference between ketamine-treated WT and GluN2CKO mice (*p* < 0.01, all cases; 99% decrease in males and 90% decrease in females compared to WT mice).

### Antidepressant-like effect

**Fig. 2:**
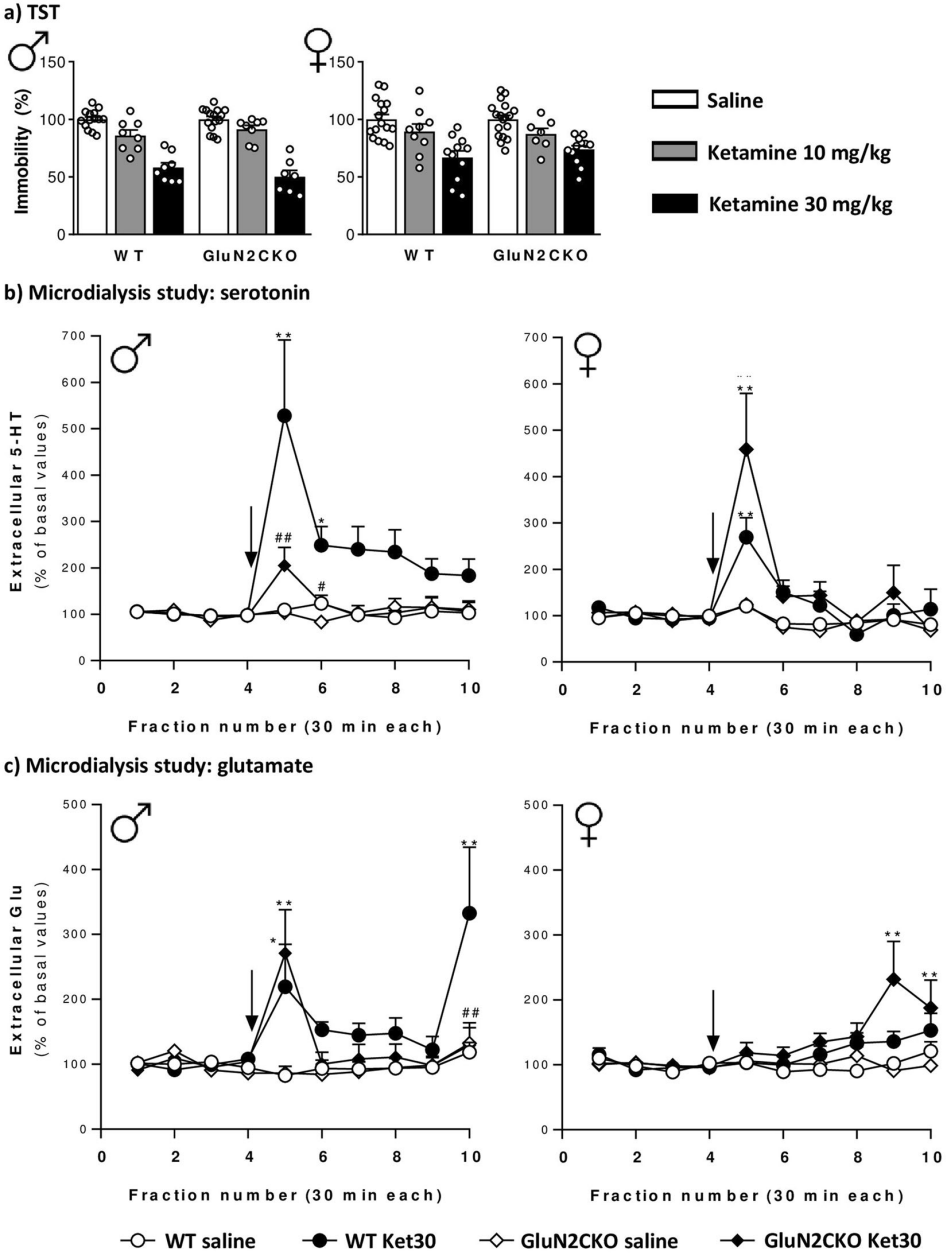
Tail suspension test and microdyalisis study in WT and GluN2CKO mice of both genders. **a** Effects of ketamine (10 and 30 mg/kg) in male (*♂*) and female (♀) WT and GluN2CKO mice in the tail-suspension test (TST). Data are expressed as percentage of immobility from saline-treated mice. Male WT saline (*n* = 14); male GluN2CKO saline (*n* = 15); female WT saline (*n* = 16); female GluN2CKO saline (*n* = 18); male WT and GluN2CKO ketamine 10 and 30 mg/kg (*n* = 8); female WT ketamine 10 mg/kg (*n* = 9); female WT ketamine 30 mg/kg (*n* = 11); female GluN2CKO ketamine 10 mg/kg (*n* = 7); female GluN2CKO ketamine 30 mg/kg (*n* = 12). **b** Time course of extracellular levels of serotonin (5-HT) in the medial prefrontal cortex (mPFC) after saline or 30 mg/kg of ketamine (Ket30) administration in male and female WT and GluN2CKO mice. Male WT and GluN2CKO saline and male GluN2CKO Ket30 (*n* = 9); male WT Ket30 (*n* = 8); female WT saline (*n* = 13); female GluN2CKO saline (*n* = 14); female WT and GluN2CKO Ket30 (*n* = 11). **c** Time course of extracellular levels of glutamate (Glu) in the mPFC after saline or 30 mg/kg of ketamine (Ket30) administration in male and female WT and GluN2CKO mice. Male WT and GluN2CKO saline and Ket30 (*n* = 9); female WT saline (*n* = 12); female GluN2CKO saline (*n* = 14); female WT Ket30 (*n* = 11); female GluN2CKO Ket30 30 (*n* = 13). Microdialysis data are expressed as percentage of four basal values (fractions 1–4). The values are expressed as mean ± SEM. The arrow represents the time of IP injection of saline or ketamine. Dialysate fractions were 30 min each. *p < 0.05, **p < 0.01 vs. saline; ^#^*p* < 0.05, ^##^p < 0.01 vs. WT (Newman–Keuls post hoc test).

In the TST, two-way ANOVA showed a main effect of treatment (*F*_2,54_ = 86.36; *p* < 0.0001), but not genotype (*F*_1,50_ = 0.06; n.s.) nor treatment × genotype interaction (*F*_2,50_ = 0.13; n.s.) for male mice. For female mice, two-way ANOVA showed a main effect of treatment (*F*_2,67_ = 22.70; *p* < 0.0001), but not genotype (*F*_1,67_ = 0.16; n.s.) nor treatment × genotype interaction (*F*_2,67_ = 0.46; n.s.) (Fig. 2a).

### In vivo intracerebral microdialysis

Baseline extracellular concentrations of 5-HT in mPFC of male WT and GluN2CKO mice were 18 ± 3 fmol/50 µl (*n* = 9) and 15 ± 3 fmol/50 µl (*n* = 9), respectively (*t*(16) = 0.58; n.s.; unpaired *t*-test). Baseline extracellular concentrations of Glu in mPFC in male WT and GluN2CKO mice were 13 ± 2 pmol/50 µl (*n* = 9) and 10 ± 1 pmol/50 µl (*n* = 9), respectively (*t*(16) = 1.36; n.s.; unpaired *t*-test). Baseline extracellular concentrations of 5-HT in mPFC in female WT and GluN2CKO mice were 21 ± 4 fmol/50 µl (*n* = 13) and 18 ± 4 fmol/50 µl (*n* = 14), respectively (*t*(24.96) = 0.47; n.s.; unpaired *t*-test with Welch’s correction). Baseline extracellular concentrations of Glu in mPFC in female WT and GluN2CKO mice were 7 ± 1 pmol/50 µl (*n* = 12) and 7 ± 1 pmol/50 µl (*n* = 14), respectively (*t*(23.56) = 0.12; n.s.; unpaired *t*-test with Welch’s correction).

The effects of ketamine (30 mg/kg) on extracellular 5-HT and Glu release are shown in Fig. 2b, c. A detailed statistical analysis (three-way ANOVA) is shown in Table S1. Ketamine induced a more consistent elevation of extracellular 5-HT than of Glu, yet with genotype-related and sex-related differences.

### *c-fos* mRNA expression

**Fig. 3:**
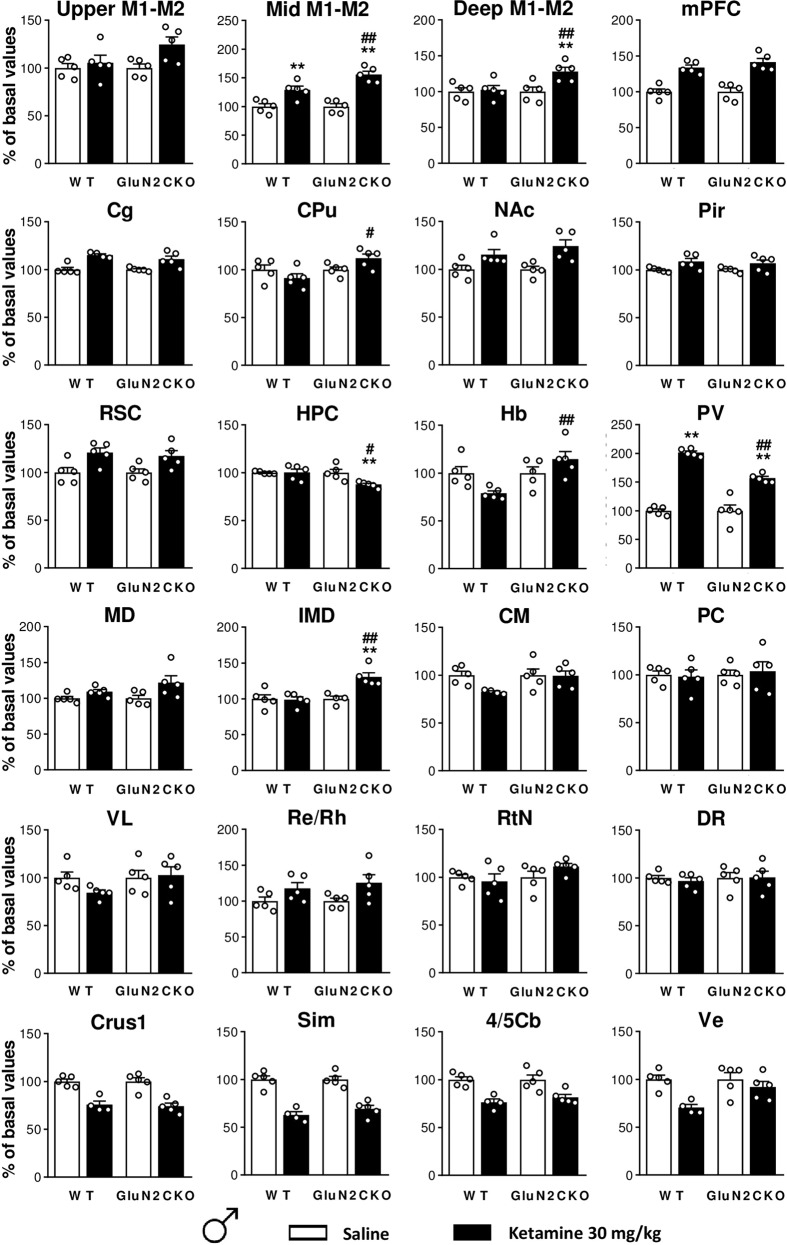
Ketamine-induced *c-fos* brain expression in male WT and GluN2CKO mice. *c-fos* mRNA expression for male (*♂*) WT and GluN2CKO mice after saline or ketamine (30 mg/kg) administration. Upper, intermediate, and deep layers of primary and secondary motor cortices (upper M1–M2, mid M1–M2, and deep M1–M2, AP: +2.10), medial prefrontal cortex (mPFC, AP: +2.10), cingulate cortex (Cg, AP: +1.18), caudate-putamen nuclei (CPu, AP: +1.18), nucleus accumbens (NAc, AP: +1.18), piriform cortex (Pir, AP: +1.18), retrosplenial cortex (RSC, AP: −1.70), hippocampus (HPC, AP: −1.70), habenula (Hb, AP: −1.70), paraventricular thalamic nucleus (PV, AP: −1.70), mediodorsal thalamic nucleus (MD, AP: −1.70), intermediodorsal thalamic nucleus (IMD, AP: −1.70), centromedial thalamic nucleus (CM, AP: −1.70), paracentral thalamic nucleus (PC, AP: −1.70), ventrolateral thalamic nucleus (VL, AP: −1.70), reuniens, and rhomboid nuclei of the thalamus (Re/Rh, AP: −1.70), reticular nucleus (RtN, AP: −1.70), dorsal raphe (DR, AP: −4.60), crus 1 of the ansiform lobule (Crus1, AP: −6.00), cerebellar simple lobule (Sim, AP: −6.00), lobules 4 and 5 of the cerebellar vermis (4/5Cb, AP: −6.00) and vestibular nucleus (Ve, AP: −6.00). *n* = 5 in all groups, except for *n* = 4 in WT ketamine groups in Cg, CM, Crus1, Sim, 4/5Cb, Ve; and *n* = 4 in GluN2CKO saline group in IMD. **p < 0.01 vs. saline; ^#^*p* < 0.05, ^##^*p* < 0.01 vs. WT (Newman–Keuls post hoc test).

**Fig. 4:**
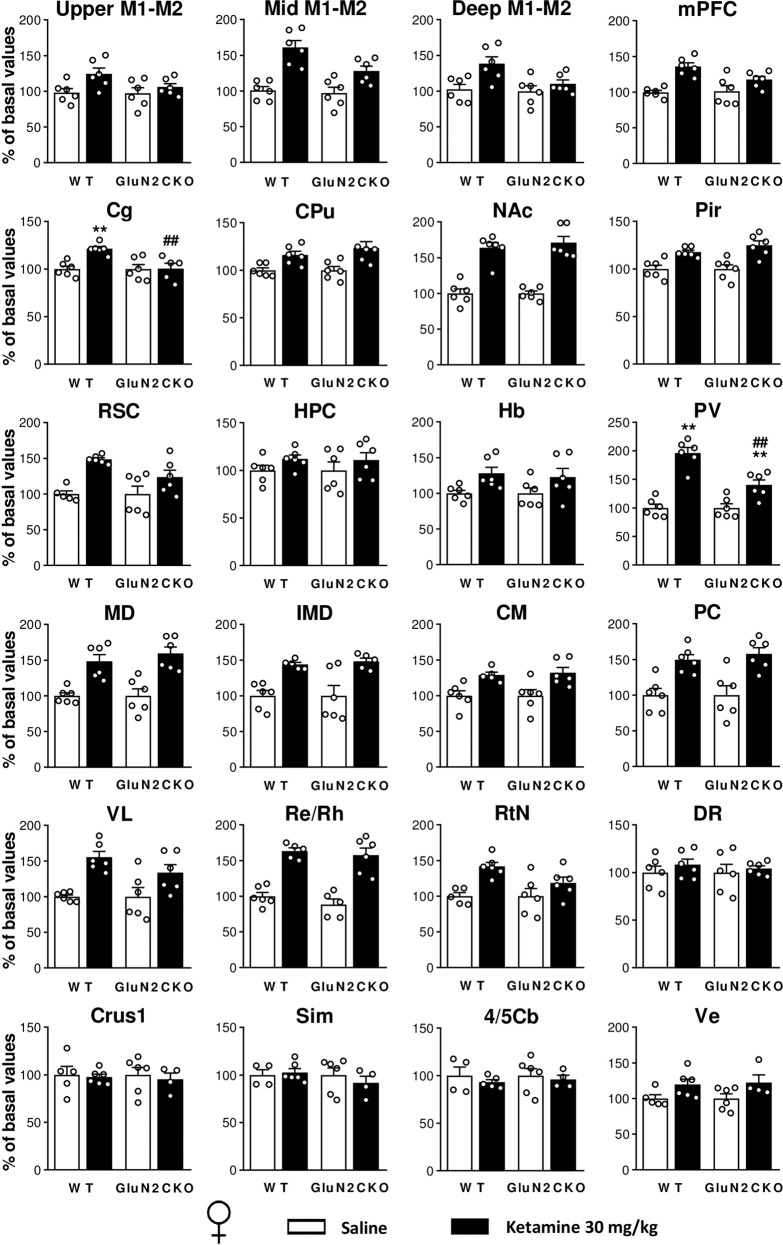
Ketamine-induced *c-fos* brain expression in female WT and GluN2CKO mice. *c-fos* mRNA expression for female (♀) WT and GluN2CKO mice after saline or ketamine (30 mg/kg) administration. Upper, intermediate, and deep layers of primary and secondary motor cortices (upper M1–M2, mid M1–M2, and deep M1–M2, AP: +2.10), medial prefrontal cortex (mPFC, AP: +2.10), cingulate cortex (Cg, AP: +1.18), caudate-putamen nuclei (CPu, AP: +1.18), nucleus accumbens (NAc, AP: +1.18), piriform cortex (Pir, AP: +1.18), retrosplenial cortex (RSC, AP: −1.70), hippocampus (HPC, AP: −1.70), habenula (Hb, AP: −1.70), paraventricular thalamic nucleus (PV, AP: −1.70), mediodorsal thalamic nucleus (MD, AP: −1.70), intermediodorsal thalamic nucleus (IMD, AP: −1.70), centromedial thalamic nucleus (CM, AP: −1.70), paracentral thalamic nucleus (PC, AP: −1.70), ventrolateral thalamic nucleus (VL, AP: −1.70), reuniens and rhomboid nuclei of the thalamus (Re/Rh, AP: −1.70), reticular nucleus (RtN, AP: −1.70), dorsal raphe (DR, AP: −4.60), crus 1 of the ansiform lobule (Crus1, AP: −6.00), cerebellar simple lobule (Sim, AP: −6.00), lobules 4 and 5 of the cerebellar vermis (4/5Cb, AP: −6.00) and vestibular nucleus (Ve, AP: −6.00). *n* = 6 in all groups, except for *n* = 5 in WT saline groups in RSC, RtN, Crus1, Ve; and *n* = 4 in WT saline groups in Sim, 4/5Cb; and *n* = 5 in GluN2CKO saline group in Re/Rh, *n* = 5 in WT ketamine groups in IMD, CM, Re/Rh, 4/5Cb; and *n* = 5 in GluN2CKO ketamine groups in Cg, IMD; and *n* = 4 in GluN2CKO ketamine groups in Crus1, Sim, Ve. **p < 0.01 vs. saline; ^##^*p* < 0.01 vs. WT (Newman–Keuls post hoc test).

The effects of ketamine (30 mg/kg) on *c-fos* mRNA expression are shown in Figs. 3, 4 and S1. A detailed statistical analysis (two-way ANOVA) is shown in Table S2. The brain structures examined are organized following a rostro-caudal axis.

In male mice, after ketamine administration there was a main effect of treatment in upper, intermediate, and deep layers of primary and secondary motor cortices (upper M1–M2, mid M1–M2, and deep M1–M2), mPFC, cingulate (Cg), nucleus accumbens (NAc), piriform cortex (Pir), retrosplenial cortex (RSC), HPC, paraventricular thalamic nucleus (PV), mediodorsal thalamic nucleus (MD), intermediodorsal thalamic nucleus (IMD), reuniens and rhomboid nuclei of the thalamus (Re/Rh), crus 1 of the ansiform lobule (Crus1), cerebellar simple lobule (Sim), lobules 4 and 5 of the cerebellar vermis (4/5Cb) and vestibular nucleus (Ve); a main effect of genotype in mid M1–M2, deep M1–M2, CPu, HPC, habenula (Hb), PV and IMD; and a main effect of treatment × genotype interaction in mid M1–M2, deep M1–M2, CPu, HPC, Hb, PV, and IMD. Post hoc comparisons performed after significant interaction between factors showed that ketamine (30 mg/kg) significantly enhanced *c-fos* mRNA expression in mid M1–M2 and PV (*p* < 0.01, all cases) in WT male mice. In male GluN2CKO mice, ketamine significantly increased *c-fos* expression in mid M1–M2, deep M1–M2, PV, IMD (*p* < 0.01, all cases). Significant differences between male WT and GluN2CKO mice treated with ketamine were found in mid M1–M2, deep M1–M2, habenula (Hb), PV, IMD (*p* < 0.01, all cases), CPu and HPC (*p* < 0.05, all cases).

In female mice, after ketamine administration there was a main effect of treatment in upper M1–M2, mid M1–M2, deep M1–M2, mPFC, Cg, CPu, NAc, Pir, RSC, Hb, PV, MD, IMD, centromedial thalamic nucleus (CM), paracentral thalamic nucleus (PC), ventrolateral thalamic nucleus (VL), Re/Rh, RtN, and Ve; a main effect of genotype in mid M1–M2, Cg and PV; and a main effect of treatment × genotype interaction in Cg and PV. Post hoc comparisons performed after significant interaction between factors showed that ketamine (30 mg/kg) significantly enhanced *c-fos* mRNA expression in Cg and PV (*p* < 0.01, all cases) in female WT mice. In female GluN2CKO mice, ketamine significantly increased *c-fos* expression in PV (*p* < 0.01). Significant differences between female WT and GluN2CKO mice treated with ketamine were found in Cg and PV (*p* < 0.01, all cases).

### fMRI: functional connectivity

**Fig. 5:**
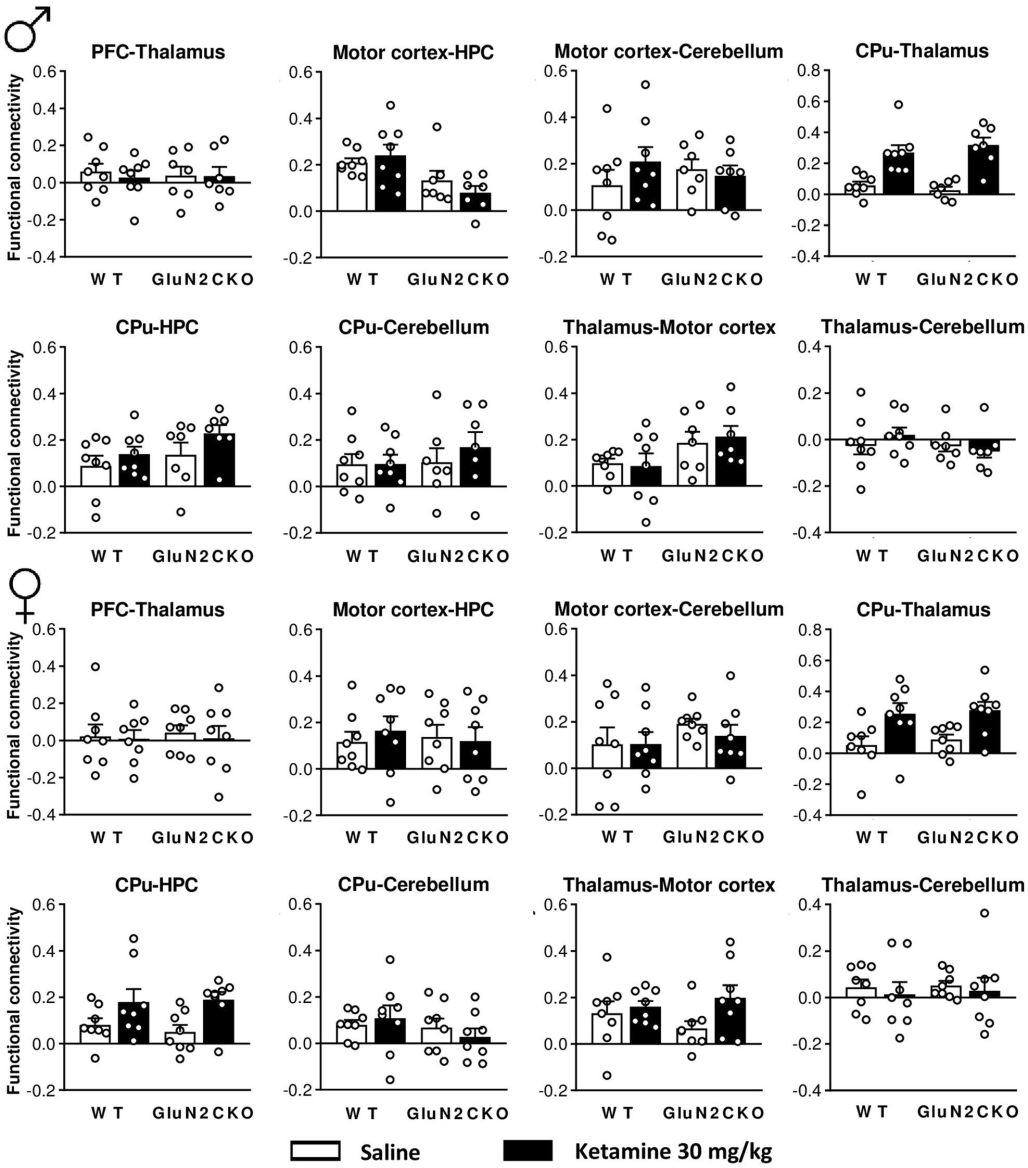
fMRI study in WT and GluN2CKO mice of both genders. Functional connectivity for male (*♂*) and female (♀) WT and GluN2CKO mice (*n* = 8 for male WT groups and for female WT and GluN2CKO groups; *n* = 7 for male GluN2CKO groups). Prefrontal cortex (PFC), hippocampus (HPC), and caudate–putamen nuclei (CPu).

The effects of ketamine (30 mg/kg) on functional connectivity are shown in Fig. 5. A detailed statistical analysis (two-way ANOVA) is shown in Table S3. In male mice, after ketamine administration there was a main effect of treatment in CPu–thalamus and CPu–motor cortex and a main effect of genotype in motor cortex–HPC connectivity. In female mice, after ketamine administration there was a main effect of treatment in PFC–CPu, PFC–HPC, CPu–thalamus, CPu–Motor cortex, CPu–HPC, and thalamus–HPC connectivity.

## Discussion

A single administration of a sub-anesthetic dose of ketamine elicits a fast-acting and sustained antidepressant action in patients with TRD. Nevertheless, its psychotomimetic effects are a significant limitation for its safe widespread use as an antidepressant. The present study shows that NMDA-Rs containing the GluN2C subunit are involved in the motor components of the psychotomimetic effects induced by ketamine but not in its antidepressant-like action. Therefore, prevention of ketamine’s effects on GluN2C subunits or the design of new agents lacking affinity for this subunit may be a step toward preventing some of the undesirable side effects of ketamine, while keeping the antidepressant response.

Here we show that ketamine induced psychotomimetic effects in both WT and GluN2CKO mice. However, stereotypes (circling) were dramatically attenuated in mice lacking the GluN2C subunit. Other motor behaviors such as ataxia (falls, hindlimb abduction) and rearings showed an improvement trend in GluN2CKO mice, suggesting a better motor coordination in the mutant mice. In agreement, acute treatment with NMDA-R antagonists like MK-801 and PCP, which precipitate a more robust behavioral syndrome than ketamine, induced less circling behavior, falls, hindlimb abduction, and increased number of rearings in GluN2CKO mice^53^, suggesting a common action of the three non-competitive NMDA-R antagonists. In addition, GluN2CKO mice showed increased latency to fall in the rotarod after PCP or MK-801 acute treatment^53^, further supporting that GluN2CKO mice exhibit a better motor coordination after NMDA-R blockade. Precisely, the improved motor coordination after ketamine treatment would explain the increased distance traveled in the OF in mutant mice. Moreover, ketamine elicited an anxiolytic-like response in WT mice as previously reported^54^, however this effect was absent in mice lacking the GluN2C subunit. Since the GluN2C subunit is highly expressed in the cerebellum and this brain structure plays a key role in motor coordination^55^, our results suggest the involvement of cerebellar GluN2C-containing NMDA-Rs in the motor components of the psychotomimetic effects induced by non-competitive NMDA-R antagonists.

Interestingly, the antidepressant-like response of ketamine was fully preserved in GluN2CKO mice. Thus, ketamine produced a similar reduction of immobility in both genotypes in the TST and FST (Fig. S2, Supplementary material). These results would indicate that the GluN2C subunit is not involved in the antidepressant-like effects of ketamine. Moreover, in our study GluN2CKO mice did not exhibit a depressive-like phenotype, as previously reported^56^, while others described basal differences in the FST^57^. These discrepancies could be explained by strain differences on anxiety-like behaviors and immobility between C57BL/6N^57^ and C57BL/6J mice (present study), as C57BL/6N mice display higher anxiety-like behavioral responses^58,59^.

Concerning the psychotomimetic and antidepressant effects of ketamine in both sexes, we did not statistically compare both male and female results because they underwent behavioral testing on alternating weeks. However, our results suggest that there are no relevant differences between males and females as similar dose-dependent effects of ketamine were found in both sexes. These results may be in contrast with previous studies in rats reporting that females were more sensitive to NMDA-R blockade by MK-801 than males, showing an increased and long-lasting recumbency^60^, locomotor activity, ataxia and head weaving^61^, and studies showing increased sensitivity to the rapid antidepressant-like effect of ketamine in females compared to their male counterparts^62,63^. In mice, ketamine exhibited controverted results in freely cycling female mice, with studies supporting this higher sensitivity^29,64^ and others reporting no differences between sexes^65^, as observed in the present study.

The present study was also aimed at investigating the neurochemical effects of acute ketamine treatment in mPFC, since this is a converging anatomical substrate for the psychotomimetic and antidepressant effects of ketamine and a relevant area in the pathophysiology of schizophrenia and depression^66,67^. NMDA-R antagonists cause net disruptions of population activity and enhance gamma and high-frequency oscillations in mPFC^26^. On the other hand, Glu and 5-HT neurotransmission in infralimbic cortex (IL, ventral subdivision of the mPFC) seems crucial for antidepressant-like responses in rodents^68,69^. Hence, an acute increase of AMPA-R-mediated glutamatergic neurotransmission in IL evoked antidepressant-like effects associated with an increased 5-HT activity^68,69^. Other studies also support the contribution of the serotonergic system in ketamine-induced antidepressant effects^44,70,71^. Thus, we evaluated ketamine-induced changes in extracellular 5-HT and Glu concentration in mPFC by in vivo microdialysis in male and female mice, since, to our knowledge, sex differences in the neurochemical effects of ketamine have been poorly addressed. An increase of extracellular levels of 5-HT were found in mPFC in WT and GluN2CKO mice of both sexes after an effective antidepressant dose of ketamine (30 mg/kg). Although some genotype differences exist, our results suggest that 5-HT may be more likely contributing to the observed antidepressant response than Glu, as no consistent increases of Glu were found in either WT or GluN2CKO female mice after ketamine administration. Interestingly, the increases in 5-HT or Glu (when found) were transient, reaching a maximum within the first 30 min after the administration of ketamine and then returning to basal levels. This temporal profile fully matches the fast pharmacokinetic profile of ketamine in male C57BL/6, which its half-life has been reported to be approximately of 13 min^72^ or between 30 and 40 min^73^. Unexpectedly, Glu levels in mPFC peaked at 2–3 h after ketamine treatment in some experimental groups, which would not be a direct effect of ketamine, given its fast-pharmacokinetic profile. Previous studies have shown that ketamine increased the extracellular levels of 5-HT in PFC of male rats^74–77^ and so did each of its enantiomers in male mice^78^. However, there are controverted results regarding Glu release in this area, with studies showing long-lasting increases of Glu^79,80^ or no effects^77^ in rats.

Altogether, our results suggest that an increase in serotonergic-but not glutamatergic-transmission may be involved in the acute antidepressant-like response of ketamine. Nevertheless, the neurochemical basis of the antidepressant response of ketamine in male and female mice deserves further attention, since sex-specific neurochemical and molecular effects of repeated ketamine have also been described^81^.

In order to identify the brain areas responding to ketamine’s treatment, we investigated (i) the expression of the immediate early gene *c-fos*, a surrogate marker of neuronal activity given its direct relationship with neuronal discharge^82–85^ and (ii) the synchronization in oxygen consumption among different brain areas by resting state fMRI studies as a measure of functional connectivity. We found synchronized changes in oxygen consumption in CPu-thalamus and CPu-motor cortex for male and female mice, an observation that may be related to the activation of the motor circuit of basal ganglia by ketamine. Interestingly, ketamine increased functional connectivity among more areas in female mice than in male mice, an outcome further supporting the sex-specific neurochemical differences we described in the microdialysis studies. However, despite the observed genotype differences in some motor behaviors, we did not find parallel differences in the functional connectivity between the cerebellum and motor cortex or motor thalamus, perhaps due to the poor resolution of fMRI analyses, unable to pick up blood flow changes in the deep cerebellar nuclei projecting to motor thalamic nuclei. Alternatively, and given the much greater neuronal discharge and neuronal package in the cerebellum, oxygen demand and consumption may be regulated in an entirely different way from the rest of the brain, which would lead to a poor functional connectivity between the cerebellum and the motor thalamus, as observed (Fig. 5).

Regarding *c-fos*, the most relevant observations in males were (i) ketamine did not systematically activate thalamic nuclei, as it has been extensively described for MK-801 and PCP treatments^25,53,86,87^ and (ii) moderate, yet consistent reductions of *c-fos* expression were found in cerebellar areas. The decreased cerebellar *c-fos* expression after ketamine is at variance with the typical drug-induced increases seen in the rest of the brain, and most likely reflects the ketamine-induced decrease of the neuronal activity of cerebellar granule cells, packed at very high density, expressing NMDA-Rs and discharging at very high rates^88^.

In females, ketamine induced a pattern of *c-fos* expression in thalamic and cerebellar areas entirely different from that seen in males. Thus, female mice did exhibit the expected increased activity in all thalamic nuclei, while no decreases in *c-fos* expression were found in the cerebellum. Genotype differences on *c-fos* expression were observed in motor cortex, CPU, HPC, Hb, PV, and IMD in male mice, while in females the differences concentrated in motor cortex, Cg, and PV. Remarkably, the dorsal raphe (DR) was not activated in male nor female mice after ketamine treatment, which would have been expected given that ketamine enhanced 5-HT neurotransmission and a previous study reported significantly increased *c-fos* immunoreactivity in DR after systemic 30 mg/kg ketamine administration^44^.

Overall, data from *c-fos* experiments suggests the involvement of cerebellar–thalamic–cortical pathways in the psychotomimetic (motor) actions of ketamine, yet with small genotype differences that do not parallel the significant differences in motor behaviors between both genotypes. The involvement of these areas agrees with the known circuitry controlling motor behavior, in which the deep cerebellar nuclei project to motor thalamic nuclei and then, to motor cortex^89–91^. The conspicuous lack of genotype differences in *c-fos* expression after ketamine suggests a comparable response of cerebellar granule cells in both genotypes. However, the limited anatomical resolution of the present histological technique did not allow assessing whether ketamine affected Purkinje cells or deep cerebellar nuclei in the same way in both genotypes, a difference that may account for behavioral differences. In addition, the intriguing sex differences observed in *c-fos* expression in cerebellar and thalamic areas suggest a differential response to ketamine of the different components of the motor circuit between male and female mice that deserves further attention.

On the other hand, antidepressant-like effects of ketamine were likely mediated by the raphe–PFC pathway, as suggested by the association between behavioral and neurochemical effects (5-HT increase), and the similar behavioral response of both genotypes to ketamine, in line with the lack of expression of GluN2C subunits in this pathway. This view is in close agreement with previous observations indicating that antidepressant effects induced by ketamine and other glutamate-based strategies depend on serotonergic activity^42,68,69^, likely involving an activation of the PFC–raphe pathway^92^ and the subsequent enhancement of serotonergic activity and 5-HT release^68,69^.

In conclusion, the present study demonstrates that GluN2C deletion allows preserving the antidepressant-like action of ketamine while reducing the psychotomimetic-associated response in mice of both sexes. Moreover, it describes dissimilar effects on ketamine-induced Glu release in mPFC and *c-fos* brain expression in male and female mice. Overall, this supports the view that glutamate-based antidepressant strategies lessening psychotomimetic actions can be developed.

## Supplementary information

Supplementary material

Table S1

Table S2

Table S3

Figure S1

Figure S2

## References

[1] Mathers CD, Loncar D (2006). Projections of global mortality and burden of disease from 2002 to 2030. PLoS Med..

[2] Kessler RC (2012). The costs of depression. Psychiatr. Clin. North Am..

[3] Trautmann S, Rehm J, Wittchen H (2016). The economic costs of mental disorders: do our societies react appropriately to the burden of mental disorders?. EMBO Rep..

[4] WHO. Depression and other common mental disorders: global health estimates. *World Health Organ.* **WHO/MSD/MER/2017.2**, 1–24 (2017).

[5] Insel T, Wang PS (2009). The STAR*D trial: revealing the need for better treatments. Psychiatr. Serv..

[6] Berman RM (2000). Antidepressant effects of ketamine in depressed patients. Biol. Psychiatry.

[7] Xu Y (2006). Effects of low-dose and very low-dose ketamine among patients with major depression: a systematic review and meta-analysis. Int. J. Neuropsychopharmacol..

[8] Zarate CA (2006). A randomized trial of an N-methyl-d-aspartate antagonist in treatment-resistant major depression. Arch. Gen. Psychiatry.

[9] Daly EJ (2018). Efficacy and safety of intranasal esketamine adjunctive to oral antidepressant therapy in treatment-resistant depression: a randomized clinical trial. JAMA Psychiatry.

[10] FDA News Release on March 5, 2019. *FDA Approves New Nasal Spray Medication for Treatment-resistant Depression*. Available Only at a Certified Doctor’s Office or Clinic. https://www.fda.gov/NewsEvents/Newsroom/PressAnnouncements/ucm632761.html (2019).

[11] Loo CK (2016). Placebo-controlled pilot trial testing dose titration and intravenous, intramuscular and subcutaneous routes for ketamine in depression. Acta Psychiatr. Scand..

[12] Romeo B, Choucha W, Fossati P, Rotge JY (2015). Meta-analysis of short- and mid-term efficacy of ketamine in unipolar and bipolar depression. Psychiatry Res..

[13] Derntl B (2019). Interaction of sex and age on the dissociative effects of ketamine action in young healthy participants. Front. Neurosci..

[14] Bartoli F (2017). Ketamine as a rapid-acting agent for suicidal ideation: a meta-analysis. Neurosci. Biobehav. Rev..

[15] Wilkinson ST (2018). The effect of a single dose of intravenous ketamine on suicidal ideation: a systematic review and individual participant data meta-analysis. Am. J. Psychiatry.

[16] Niciu MJ (2014). Clinical predictors of ketamine response in treatment-resistant major depression. J. Clin. Psychiatry.

[17] Sanacora G (2017). A consensus statement on the use of ketamine in the treatment of mood disorders. JAMA Psychiatry.

[18] Zanos P, Gould TD (2018). Mechanisms of ketamine action as an antidepressant. Mol. Psychiatry.

[19] Homayoun H, Moghaddam B (2007). NMDA receptor hypofunction produces opposite effects on prefrontal cortex interneurons and pyramidal neurons. J. Neurosci..

[20] Tsai G, Coyle JT (2002). Glutamatergic mechanisms in schizophrenia. Annu. Rev. Pharmacol. Toxicol..

[21] Amat-Foraster M (2018). Temporally dissociable effects of ketamine on neuronal discharge and gamma oscillations in rat thalamo-cortical networks. Neuropharmacology.

[22] Troyano-Rodriguez E (2014). Phencyclidine inhibits the activity of thalamic reticular gamma-aminobutyric acidergic neurons in rat brain. Biol. Psychiatry.

[23] Celada P (2013). Disruption of thalamocortical activity in schizophrenia models: Relevance to antipsychotic drug action. Int. J. Neuropsychopharmacol..

[24] Kargieman L, Santana N, Mengod G, Celada P, Artigas F (2007). Antipsychotic drugs reverse the disruption in prefrontal cortex function produced by NMDA receptor blockade with phencyclidine. Proc. Natl Acad. Sci. USA.

[25] Santana N, Troyano-Rodriguez E, Mengod G, Celada P, Artigas F (2011). Activation of thalamocortical networks by the N-methyl-d-aspartate receptor antagonist phencyclidine: reversal by clozapine. Biol. Psychiatry.

[26] Amat-Foraster M (2019). Modulation of thalamo-cortical activity by the NMDA receptor antagonists ketamine and phencyclidine in the awake freely-moving rat. Neuropharmacology.

[27] Lladó-Pelfort L (2016). Phencyclidine-induced disruption of oscillatory activity in prefrontal cortex: effects of antipsychotic drugs and receptor ligands. Eur. Neuropshychopharmacol..

[28] van den Munkhof HE, Arnt J, Celada P, Artigas F (2017). The antipsychotic drug brexpiprazole reverses phencyclidine-induced disruptions of thalamocortical networks. Eur. Neuropsychopharmacol..

[29] Zanos P (2016). NMDAR inhibition-independent antidepressant actions of ketamine metabolites. Nature.

[30] Li N (2010). mTOR-dependent synapse formation underlies the rapid antidepressant effects of NMDA antagonists. Science.

[31] Zhou W (2014). Ketamine-induced antidepressant effects are associated with AMPA receptors-mediated upregulation of mTOR and BDNF in rat hippocampus and prefrontal cortex. Eur. Psychiatry.

[32] Abdallah CG (2020). (2R,6R)-Hydroxynorketamine (HNK) plasma level predicts poor antidepressant response: is this the end of the HNK pipeline?. Neuropsychopharmacology.

[33] Kotermanski SE, Johnson JW (2009). Mg2+imparts NMDA receptor subtype selectivity to the Alzheimer’s drug memantine. J. Neurosci..

[34] Khlestova E, Johnson JW, Krystal JH, Lisman J (2016). The role of GluN2C-containing NMDA receptors in ketamine’s psychotogenic action and in schizophrenia models. J. Neurosci..

[35] Farrant M, Feldmeyer D, Takahashi T, Cull-Candy SG (1994). NMDA-receptor channel diversity in the developing cerebellum. Nature.

[36] Karavanova I, Vasudevan K, Cheng J, Buonanno A (2007). Novel regional and developmental NMDA receptor expression patterns uncovered in NR2C subunit-β-galactosidase knock-in mice. Mol. Cell. Neurosci..

[37] Monyer H, Burnashev N, Laurie DJ, Sakmann B, Seeburg PH (1994). Developmental and regional expression in the rat brain and functional properties of four NMDA receptors. Neuron.

[38] Wenzel A, Fritschy JM, Mohler H, Benke D (1997). NMDA receptor heterogeneity during postnatal development of the rat brain: differential expression of the NR2A, NR2B, and NR2C subunit proteins. J. Neurochem..

[39] Ravikrishnan A (2018). Region-specific expression of NMDA receptor GluN2C subunit in parvalbumin-positive neurons and astrocytes: analysis of GluN2C expression using a novel reporter model. Neuroscience.

[40] Zhang Y, Llinas RR, Lisman JE (2009). Inhibition of NMDARs in the nucleus reticularis of the thalamus produces delta frequency bursting. Front. Neural Circuits.

[41] Zhang Y, Buonanno A, Vertes RP, Hoover WB, Lisman JE (2012). NR2C in the thalamic reticular nucleus; effects of the NR2C knockout. PLoS ONE.

[42] Ebralidze AK, Rossi DJ, Tonegawa S, Slater T (1996). Modification of NMDA receptor channels and synaptic transmission by targeted disruption of the NR2C gene. J. Neurosci..

[43] Cathala L, Misra C, Cull-Candy S (2000). Developmental profile of the changing properties of NMDA receptors at cerebellar mossy fiber-granule cell synapses. J. Neurosci..

[44] Albert PR (2015). Why is depression more prevalent in women?. J. Psychiatry Neurosci..

[45] Fukumoto K, Iijima M, Chaki S (2016). The antidepressant effects of an mGlu2/3 receptor antagonist and ketamine require AMPA receptor stimulation in the mPFC and subsequent activation of the 5-HT neurons in the DRN. Neuropsychopharmacology.

[46] Scorza MC, Castañé A, Bortolozzi A, Artigas F (2010). Clozapine does not require 5-HT1A receptors to block the locomotor hyperactivity induced by MK-801. Clz and MK-801 in KO1A mice. Neuropharmacology.

[47] Spanos LJ, Yamamoto BK (1989). Acute and subchronic effects of methylenedioxymethamphetamine [(+/-)MDMA] on locomotion and serotonin syndrome behavior in the rat. Pharmacol. Biochem. Behav..

[48] Castañé A, Artigas F, Bortolozzi A (2008). The absence of 5-HT1A receptors has minor effects on dopamine but not serotonin release evoked by MK-801 in mice prefrontal cortex. Psychopharmacology.

[49] López-Gil X, Artigas F, Adell A (2009). Role of different monoamine receptors controlling MK-801-induced release of serotonin and glutamate in the medial prefrontal cortex: relevance for antipsychotic action. Int. J. Neuropsychopharmacol..

[50] Santana N, Bortolozzi A, Serrats J, Mengod G, Artigas F (2004). Expression of serotonin1A and serotonin2A receptors in pyramidal and GABAergic neurons of the rat prefrontal cortex. Cereb. Cortex.

[51] Franklin K. B. J. & Paxinos G. *The Mouse Brain in Stereotaxi Coordinates*, 3rd edn. (Elsevier Academic Press, London, 2007).

[52] Otsu N (1979). A threshold selection method from gray-level histograms. IEEE Trans. Syst. Man Cybern..

[53] Tarrés-Gatius, M. et al. Involvement of the GluN2C subunit in the behavioural syndrome induced by non-competitive NMDA antagonists. *Eur. Neuropsychopharmacol.* **28**, abstr. S37 (2018).

[54] Fraga DB (2018). Anxiolytic effects of ascorbic acid and ketamine in mice. J. Psychiatr. Res..

[55] Manto M (2012). Consensus paper: roles of the cerebellum in motor control—the diversity of ideas on cerebellar involvement in movement. Cerebellum.

[56] Hillman BG, Gupta SC, Stairs DJ, Buonanno A, Dravid SM (2011). Behavioral analysis of NR2C knockout mouse reveals deficit in acquisition of conditioned fear and working memory. Neurobiol. Learn. Mem..

[57] Shelkar GP (2019). Differential effect of NMDA receptor GluN2C and GluN2D subunit ablation on behavior and channel blocker-induced schizophrenia phenotypes. Sci. Rep..

[58] Matsuo N (2010). Behavioral profiles of three C57BL/6 substrains. Front. Behav. Neurosci..

[59] Simon MM (2013). A comparative phenotypic and genomic analysis of C57BL/6J and C57BL/6N mouse strains. Genome Biol..

[60] Hur GH, Son WC, Shin S, Kang JK, Kim YB (1999). Sex differences in dizocilpine (MK-801) neurotoxicity in rats. Environ. Toxicol. Pharmacol..

[61] Hönack D, Löscher W (1993). Sex differences in NMDA receptor mediated responses in rats. Brain Res..

[62] Carrier N, Kabbaj M (2013). Sex differences in the antidepressant-like effects of ketamine. Neuropharmacology.

[63] Sarkar A, Kabbaj M (2016). Sex differences in effects of ketamine on behavior, spine density, and synaptic proteins in socially isolated rats. Biol. Psychiatry.

[64] Franceschelli A, Sens J, Herchick S, Thelen C, Pitychoutis PM (2015). Sex differences in the rapid and the sustained antidepressant-like effects of ketamine in stress-naïve and ‘depressed’ mice exposed to chronic mild stress. Neuroscience.

[65] Dossat AM, Wright KN, Strong CE, Kabbaj M (2018). Behavioral and biochemical sensitivity to low doses of ketamine: influence of estrous cycle in C57BL/6 mice. Neuropharmacology.

[66] Davey CG, Breakspear M, Pujol J, Harrison BJ (2017). A brain model of disturbed self-appraisal in depression. Am. J. Psychiatry.

[67] Pomarol-Clotet E (2010). Medial prefrontal cortex pathology in schizophrenia as revealed by convergent findings from multimodal imaging. Mol. Psychiatry.

[68] Gasull-Camós J, Tarrés-Gatius M, Artigas F, Castañé A (2017). Glial GLT-1 blockade in infralimbic cortex as a new strategy to evoke rapid antidepressant-like effects in rats. Transl. Psychiatry.

[69] Gasull-Camós J (2018). Serotonergic mechanisms involved in antidepressant-like responses evoked by GLT-1 blockade in rat infralimbic cortex. Neuropharmacology.

[70] Gigliucci V (2013). Ketamine elicits sustained antidepressant-like activity via a serotonin-dependent mechanism. Psychopharmacology.

[71] Pham TH (2017). Ketamine treatment involves medial prefrontal cortex serotonin to induce a rapid antidepressant-like activity in BALB/cJ mice. Neuropharmacology.

[72] Maxwell CR (2006). Ketamine produces lasting disruptions in encoding of sensory stimuli. J. Pharmacol. Exp. Ther..

[73] Sato Y (2004). Chronopharmacological studies of ketamine in normal and NMDA E1 receptor knockout mice. Br. J. Anaesth..

[74] Amargós-Bosch M, López-Gil X, Artigas F, Adell A (2006). Clozapine and olanzapine, but not haloperidol, suppress serotonin efflux in the medial prefrontal cortex elicited by phencyclidine and ketamine. Int. J. Neuropsychopharmacol..

[75] Kinoshita H (2018). Ketamine-induced prefrontal serotonin release is mediated by cholinergic neurons in the pedunculopontine tegmental nucleus. Int. J. Neuropsychopharmacol..

[76] López-Gil X (2012). Importance of inter-hemispheric prefrontal connection in the effects of non-competitive NMDA receptor antagonists. Int. J. Neuropsychopharmacol..

[77] López-Gil X (2019). Role of serotonin and noradrenaline in the rapid antidepressant action of ketamine. ACS Chem. Neurosci..

[78] Ago Y (2019). (R)-ketamine induces a greater increase in prefrontal 5-HT release than (S)-ketamine and ketamine metabolites via an AMPA receptor-independent mechanism. Int. J. Neuropsychopharmacol..

[79] Moghaddam B, Adams BW, Verma A, Daly D (1997). Activation of glutamatergic neurotransmission by ketamine: a novel step in the pathway from NMDA receptor blockade to dopaminergic and cognitive disruptions associated with the prefrontal cortex. J. Neurosci..

[80] Lorrain DS, Baccei CS, Bristow LJ, Anderson JJ, Varney MA (2003). Effects of ketamine and N-methyl-d-aspartate on glutamate and dopamine release in the rat prefrontal cortex: Modulation by a group II selective metabotropic glutamate receptor agonist LY379268. Neuroscience.

[81] Thelen C, Sens J, Mauch J, Pandit R, Pitychoutis PM (2016). Repeated ketamine treatment induces sex-specific behavioral and neurochemical effects in mice. Behav. Brain Res..

[82] Dragunow M, Faull R (1989). The use of c-fos as a metabolic marker in neuronal pathway tracing. J. Neurosci. Methods.

[83] Konkle ATM, Bielajew C (2004). Tracing the neuroanatomical profiles of reward pathways with markers of neuronal activation. Rev. Neurosci..

[84] Kovács KJ (2008). Measurement of immediate-early gene activation-c-fos and beyond. J. Neuroendocrinol..

[85] Lladó-Pelfort L, Santana N, Ghisi V, Artigas F, Celada P (2012). 5-HT1A receptor agonists enhance pyramidal cell firing in prefrontal cortex through a preferential action on GABA interneurons. Cereb. Cortex.

[86] Castañé A, Santana N, Artigas F (2015). PCP-based mice models of schizophrenia: differential behavioral, neurochemical and cellular effects of acute and subchronic treatments. Psychopharmacology.

[87] Inta D, Trusel M, Riva MA, Sprengel R, Gass P (2009). Differential c-Fos induction by different NMDA receptor antagonists with antidepressant efficacy: potential clinical implications. Int. J. Neuropsychopharmacol..

[88] van Beugen BJ, Gao Z, Boele HJ, Hoebeek F, De Zeeuw CI (2013). High frequency burst firing of granule cells ensures transmission at the parallel fiber to purkinje cell synapse at the cost of temporal coding. Front. Neural Circuits.

[89] Dum RP, Strick PL (2003). An unfolded map of the cerebellar dentate nucleus and its projections to the cerebral cortex. J. Neurophysiol..

[90] Kuramoto E (2009). Two types of thalamocortical projections from the motor thalamic nuclei of the rat: a single neuron-tracing study using viral vectors. Cereb. Cortex.

[91] Percheron G, Franqois C, Talbi B, Yelnik J, Ffnelon G (1996). The primate motor thalamus. Brain Res. Rev..

[92] Fuchikami (2015). Optogenetic stimulation of infralimbic PFC reproduces ketamine’s rapid and sustained antidepressant actions. PNAS.

