# Supplementary material for: Involvement of NMDA receptors containing the GluN2C subunit in the psychotomimetic and antidepressant-like effects of ketamine
Source: Transl Psychiatry. 2020 Dec 10;10:427. doi: 10.1038/s41398-020-01110-y (PMC7729946; doi:10.1038/s41398-020-01110-y)
Supplement: Supplementary file 1 — Supplementary material [file 41398_2020_1110_MOESM1_ESM.docx]

**Supplementary material**

**Methods**

Forced swim test (FST). Mice were tested 30 min after ketamine/saline administration. They were forced to swim in a clear methacrylate cylinder (15 cm diameter x 30 cm height) containing 20 cm of water maintained at 24 ± 1°C, as described by Porsolt *et al*. (*Eur. J. Pharmacol*. **51**, 291–294 (1978)). The cylinder was filled with fresh water for every single animal. Mice were exposed to a 6-min single trial, which was recorded using a video camera system. Subsequently, video recordings were analyzed by an experimenter blind to mice genotype and treatment, and the immobility time was quantified in the last 4 min of the test.

**Statistical analysis**

The data are expressed as mean ± S.E.M.. Statistical analysis was carried out using Kruskal-Wallis test followed by Dunn’s *post hoc* comparisons. In all cases, the level of significance was set at p<0.05.

**Results**

In the male FST, ketamine (30 mg/kg) reduced the immobility time in both genotypes (Kruskal-Wallis test=25.22; p=0.0001) (figure S2).

**Figure legends**

**Figure S1.** Representative film images of *c-fos* mRNA expression in male (♂) and female (♀) WT and GluN2CKO mice after saline or ketamine (30 mg/kg) administration.

**Figure S2.** Effects of ketamine (10 and 30 mg/kg) in male WT and GluN2CKO mice in the forced swimming test (FST). Data are expressed as percentage (%) of immobility from saline-treated mice. WT saline (n=14); GluN2CKO saline (n=15); WT and GluN2CKO ketamine 10 (n=8); WT ketamine 30 (n=4); GluN2CKO ketamine 30 (n=7). **p<0.01 *vs* saline (Dunn’s *post hoc* test). ketamine (30 mg/kg)-treated WT mice showed bad motor coordination and high risk of drowning. Therefore, only in this group, the number of animals was reduced to 4, for ethical reasons.
