# Supplementary material for: Involvement of NMDA receptors containing the GluN2C subunit in the psychotomimetic and antidepressant-like effects of ketamine
Source: Transl Psychiatry. 2020 Dec 10;10:427. doi: 10.1038/s41398-020-01110-y (PMC7729946; doi:10.1038/s41398-020-01110-y)
Supplement: Supplementary file 2 — Table S1 [file 41398_2020_1110_MOESM2_ESM.docx]

|  |  | **Treatment (T)** | | **Genotype (G)** | | **Time (t)** | | **T x G** | | **T x t** | | | **G x t** | | | **T x G x t** | | |
| --- | --- | --- | --- | --- | --- | --- | --- | --- | --- | --- | --- | --- | --- | --- | --- | --- | --- | --- |
| **♂** | **5-HT** | F_1,31_=19.17 | p<0.0001 | F_1,31_=12.01 | p<0.01 | F_9,279_=8.33 | p<0.0000 | F_1,31_=11.81 | p<0.01 | | F_9,279_=7.57 | p<0.0000 | | F_9,279_=3.37 | p<0.001 | | F_9,279_=3.11 | p<0.01 |
|  | **Glu** | F_1,32_=16.69 | p<0.001 | F_1,32_=2.40 | n.s. | F_9,288_=5.44 | p<0.0000 | F_1,32_=2.67 | n.s. | | F_9,288_=4.71 | p<0.0000 | | F_9,288_=1.77 | n.s. | | F_9,288_=2.01 | p<0.05 |
| **♀** | **5-HT** | F_1,46_=8.18 | p<0.01 | F_1,46_=2.05 | n.s. | F_9,414_=7.74 | p<0.0000 | F_1,46_=2.38 | n.s. | | F_9,414_=5.55 | p<0.0000 | | F_9,414_=1.56 | n.s. | | F_9,414_=1.39 | n.s. |
|  | **Glu** | F_1,46_=11.41 | p<0.01 | F_1,46_=2.23 | n.s. | F_9,414_=5.00 | p<0.0000 | F_1,46_=1.69 | n.s. | | F_9,414_=4.24 | p<0.0000 | | F_9,414_=0.89 | n.s. | | F_9,414_=1.56 | n.s. |

**Table S1.** Three-way ANOVA (treatment (T), genotype (G) and time (t) as factors) for the extracellular levels of serotonin (5-HT) and glutamate (Glu) in the medial prefrontal cortex (mPFC) of male (♂) and female (♀) WT and GluN2CKO mice after the acute administration of ketamine (30 mg/kg).
