# Supplementary material for: Involvement of NMDA receptors containing the GluN2C subunit in the psychotomimetic and antidepressant-like effects of ketamine
Source: Transl Psychiatry. 2020 Dec 10;10:427. doi: 10.1038/s41398-020-01110-y (PMC7729946; doi:10.1038/s41398-020-01110-y)
Supplement: Supplementary file 3 — Table S2 [file 41398_2020_1110_MOESM3_ESM.docx]

|  | **♂ Treatment (T)** | | **Genotype (G)** | | **T x G** | | **♀ Treatment (T)** | | **Genotype (G)** | | **T x G** | |
| --- | --- | --- | --- | --- | --- | --- | --- | --- | --- | --- | --- | --- |
| **Upper M1-M2** | F_1,16_=5.43 | p<0.05 | F_1,16_=2.20 | n.s. | F_1,16_=2.20 | n.s. | F_1,20_=6.69 | p<0.05 | F_1,20_=2.07 | n.s. | F_1,20_=1.69 | n.s. |
| **Mid M1-M2** | F_1,16_=55.48 | p<0.0001 | F_1,16_=5.52 | p<0.05 | F_1,16_=5.52 | p<0.05 | F_1,20_=34.84 | p<0.0001 | F_1,20_=5.63 | p<0.05 | F_1,20_=3.64 | n.s. |
| **Deep M1-M2** | F_1,16_=6.62 | p<0.05 | F_1,16_=4.70 | p<0.05 | F_1,16_=4.70 | p<0.05 | F_1,20_=9.14 | p<0.01 | F_1,20_=4.07 | n.s. | F_1,20_=2.75 | n.s. |
| **mPFC** | F_1,16_=67,81 | p<0.0001 | F_1,16_=0.71 | n.s. | F_1,16_=0.71 | n.s. | F_1,20_=23.62 | p<0.0001 | F_1,20_=2.47 | n.s. | F_1,20_=3.32 | n.s. |
| **Cg** | F_1,15_=31.27 | p<0.0001 | F_1,15_=0.91 | n.s. | F_1,15_=0.91 | n.s. | F_1,19_=7.44 | p<0.05 | F_1,19_=6.77 | p<0.05 | F_1,19_=6.77 | p<0.05 |
| **CPu** | F_1,16_=0.71 | n.s. | F_1,16_=5.93 | p<0.05 | F_1,16_=5.93 | p<0.05 | F_1,20_=17.98 | p<0.001 | F_1,20_=0.61 | n.s. | F_1,20_=0.61 | n.s. |
| **NAc** | F_1,16_=15.60 | p<0.01 | F_1,16_=0.81 | n.s. | F_1,16_=0.81 | n.s. | F_1,20_=97.84 | p<0.0001 | F_1,20_=0.27 | n.s. | F_1,20_=0.27 | n.s. |
| **Pir** | F_1,16_=8.48 | p<0.05 | F_1,16_=0.13 | n.s. | F_1,16_=0.13 | n.s. | F_1,20_=28.70 | p<0.0001 | F_1,20_=0.80 | n.s. | F_1,20_=0.80 | n.s. |
| **RSC** | F_1,16_=14.54 | p<0.01 | F_1,16_=0.13 | n.s. | F_1,16_=0.13 | n.s. | F_1,19_=19.91 | p<0.001 | F_1,19_=2.40 | n.s. | F_1,19_=2.40 | n.s. |
| **HPC** | F_1,16_=5.47 | p<0.05 | F_1,16_=5.95 | p<0.05 | F_1,16_=5.95 | p<0.05 | F_1,20_=2.88 | n.s. | F_1,20_=0.01 | n.s. | F_1,20_=0.01 | n.s. |
| **Hb** | F_1,16_=0.29 | n.s. | F_1,16_=7.98 | p<0.05 | F_1,16_=7.98 | p<0.05 | F_1,20_=8.68 | p<0.01 | F_1,20_=0.09 | n.s. | F_1,20_=0.09 | n.s. |
| **PV** | F_1,16_=187.30 | p<0.0001 | F_1,16_=15.04 | p<0.01 | F_1,16_=15.04 | p<0.01 | F_1,20_=69.96 | p<0.0001 | F_1,20_=11.69 | p<0.01 | F_1,20_=11.69 | p<0.01 |
| **MD** | F_1,16_=7.39 | p<0.05 | F_1,16_=1.23 | n.s. | F_1,16_=1.23 | n.s. | F_1,20_=40.03 | p<0.0001 | F_1,20_=0.41 | n.s. | F_1,20_=0.41 | n.s. |
| **IMD** | F_1,15_=8.02 | p<0.05 | F_1,15_=9.48 | p<0.01 | F_1,15_=9.48 | p<0.01 | F_1,18_=24.34 | p<0.0001 | F_1,18_=0.05 | n.s. | F_1,18_=0.05 | n.s. |
| **CM** | F_1,15_=3.33 | n.s. | F_1,15_=2.74 | n.s. | F_1,15_=2.74 | n.s. | F_1,19_=18.25 | p<0.001 | F_1,19_=0.05 | n.s. | F_1,19_=0.05 | n.s. |
| **PC** | F_1,16_=0.02 | n.s. | F_1,16_=0.18 | n.s. | F_1,16_=0.18 | n.s. | F_1,20_=29.22 | p<0.0001 | F_1,20_=0.18 | n.s. | F_1,20_=0.18 | n.s. |
| **VL** | F_1,16_=0.93 | n.s. | F_1,16_=1.84 | n.s. | F_1,16_=1.84 | n.s. | F_1,20_=21.91 | p<0.001 | F_1,20_=1.29 | n.s. | F_1,20_=1.29 | n.s. |
| **Re/Rh** | F_1,16_=7.57 | p<0.05 | F_1,16_=0.24 | n.s. | F_1,16_=0.24 | n.s. | F_1,18_=76.42 | p<0.0001 | F_1,18_=1.30 | n.s. | F_1,18_=0.15 | n.s. |
| **RtN** | F_1,16_=0.39 | n.s. | F_1,16_=2.03 | n.s. | F_1,16_=2.03 | n.s. | F_1,19_=14.36 | p<0.01 | F_1,19_=2.10 | n.s. | F_1,19_=2.10 | n.s. |
| **DR** | F_1,16_=0.09 | n.s. | F_1,16_=0.14 | n.s. | F_1,16_=0.14 | n.s. | F_1,20_=0.92 | n.s. | F_1,20_=0.10 | n.s. | F_1,20_=0.10 | n.s. |
| **Crus1** | F_1,15_=54.79 | p<0.0001 | F_1,15_=0.05 | n.s. | F_1,15_=0.05 | n.s. | F_1,17_=0.27 | n.s. | F_1,17_=0.03 | n.s. | F_1,17_=0.03 | n.s. |
| **Sim** | F_1,15_=91.17 | p<0.0001 | F_1,15_=0.80 | n.s. | F_1,15_=0.80 | n.s. | F_1,16_=0.19 | n.s. | F_1,16_=0.72 | n.s. | F_1,16_=0.72 | n.s. |
| **4/5Cb** | F_1,15_=30.77 | p<0.0001 | F_1,15_=0.49 | n.s. | F_1,15_=0.49 | n.s. | F_1,15_=0.68 | n.s. | F_1,15_=0.05 | n.s. | F_1,15_=0.05 | n.s. |
| **Ve** | F_1,15_=11.44 | p<0.01 | F_1,15_=3.72 | n.s. | F_1,15_=3.72 | n.s. | F_1,17_=7.85 | p<0.05 | F_1,17_=0.04 | n.s. | F_1,17_=0.04 | n.s. |

**Table S2.** Two-way ANOVA (treatment (T) and genotype (G) as factors) for *c-fos* expression data in male (♂) and female (♀) WT and GluN2CKO mice. Upper, intermediate and deep layers of primary and secondary motor cortices (Upper M1-M2, Mid M1-M2 and Deep M1-M2, AP: +2.10), medial prefrontal cortex (mPFC, AP: +2.10), cingulate cortex (Cg, AP: +1.18), caudate-putamen nuclei (CPu, AP: +1.18), nucleus accumbens (NAc, AP: +1.18), piriform cortex (Pir, AP: +1.18), retrosplenial cortex (RSC, AP: -1.70), hippocampus (HPC, AP: -1.70), habenula (Hb, AP: -1.70), paraventricular thalamic nucleus (PV, AP: -1.70), mediodorsal thalamic nucleus (MD, AP: -1.70), intermediodorsal thalamic nucleus (IMD, AP: -1.70), centromedial thalamic nucleus (CM, AP: -1.70), paracentral thalamic nucleus (PC, AP: -1.70), ventrolateral thalamic nucleus (VL, AP: -1.70), reuniens and rhomboid nuclei of the thalamus (Re/Rh, AP: -1.70), reticular nucleus (RtN, AP: -1.70), dorsal raphe (DR, AP: -4.60), crus 1 of the ansiform lobule (Crus1, AP: -6.00), cerebellar simple lobule (Sim, AP: -6.00), lobules 4 and 5 of the cerebellar vermis (4/5Cb, AP: -6.00) and vestibular nucleus (Ve, AP: -6.00).
