# Supplementary material for: Involvement of NMDA receptors containing the GluN2C subunit in the psychotomimetic and antidepressant-like effects of ketamine
Source: Transl Psychiatry. 2020 Dec 10;10:427. doi: 10.1038/s41398-020-01110-y (PMC7729946; doi:10.1038/s41398-020-01110-y)
Supplement: Supplementary file 4 — Table S3 [file 41398_2020_1110_MOESM4_ESM.docx]

|  | **♂ Treatment (T)** | |  | | **Genotype (G)** | | |  | | **T x G** | | **♀ Treatment (T)** | |  | **Genotype (G)** | | | |  | | **T x G** | | |
| --- | --- | --- | --- | --- | --- | --- | --- | --- | --- | --- | --- | --- | --- | --- | --- | --- | --- | --- | --- | --- | --- | --- | --- |
| **PFC-CPu** | F_1,13_=1.87 | n.s. |  | F_1,13_=0.11 | | n.s. |  | | F_1,13_=0.87 | | n.s. | F_1,14_=7.42 | p<0.05 | | |  | F_1,14_=0.82 | n.s. | |  | | F_1,14_=0.67 | n.s. |
| **PFC-Thalamus** | F_1,13_=0.17 | n.s. |  | F_1,13_=0.03 | | n.s. |  | | F_1,13_=0.11 | | n.s. | F_1,14_=0.17 | n.s. | | |  | F_1,14_=0.03 | n.s. | |  | | F_1,14_=0.03 | n.s. |
| **PFC-Motor cortex** | F_1,13_=3.98 | n.s. |  | F_1,13_=0.00 | | n.s. |  | | F_1,13_=3.93 | | n.s. | F_1,14_=0.50 | n.s. | | |  | F_1,14_=0.88 | n.s. | |  | | F_1,14_=0.48 | n.s. |
| **PFC-Cerebellum** | F_1,13_=1.11 | n.s. |  | F_1,13_=0.08 | | n.s. |  | | F_1,13_=0.95 | | n.s. | F_1,14_=0.44 | n.s. | | |  | F_1,14_=0.57 | n.s. | |  | | F_1,14_=4.36 | n.s. |
| **PFC-HPC** | F_1,13_=0.47 | n.s. |  | F_1,13_=2.68 | | n.s. |  | | F_1,13_=0.95 | | n.s. | F_1,14_=4.70 | p<0.05 | | |  | F_1,14_=0.04 | n.s. | |  | | F_1,14_=1.01 | n.s. |
| **CPu-Thalamus** | F_1,13_=48.57 | p<0.0001 |  | F_1,13_=0.05 | | n.s. |  | | F_1,13_=1.17 | | n.s. | F_1,14_=31.83 | p<0.0001 | | |  | F_1,14_=0.17 | n.s. | |  | | F_1,14_=0.03 | n.s. |
| **CPu-Motor cortex** | F_1,13_=7.43 | p<0.05 |  | F_1,13_=0.48 | | n.s. |  | | F_1,13_=0.86 | | n.s. | F_1,14_=6.44 | p<0.05 | | |  | F_1,14_=1.75 | n.s. | |  | | F_1,14_=0.57 | n.s. |
| **CPu-Cerebellum** | F_1,13_=1.13 | n.s. |  | F_1,13_=0.35 | | n.s. |  | | F_1,13_=1.04 | | n.s. | F_1,14_=0.02 | n.s. | | |  | F_1,14_=1.57 | n.s. | |  | | F_1,14_=0.63 | n.s. |
| **CPu-HPC** | F_1,13_=4.56 | n.s. |  | F_1,13_=1.91 | | n.s. |  | | F_1,13_=0.38 | | n.s. | F_1,14_=8.52 | p<0.05 | | |  | F_1,14_=0.08 | n.s. | |  | | F_1,14_=0.25 | n.s. |
| **Thalamus-Motor cortex** | F_1,13_=0.06 | n.s. |  | F_1,13_=3.82 | | n.s. |  | | F_1,13_=0.46 | | n.s. | F_1,14_=4.35 | n.s. | | |  | F_1,14_=0.07 | n.s. | |  | | F_1,14_=1.88 | n.s. |
| **Thalamus-Cerebellum** | F_1,13_=0.06 | n.s. |  | F_1,13_=0.90 | | n.s. |  | | F_1,13_=0.69 | | n.s. | F_1,14_=0.48 | n.s. | | |  | F_1,14_=0.06 | n.s. | |  | | F_1,14_=0.01 | n.s. |
| **Thalamus-HPC** | F_1,13_=0.01 | n.s. |  | F_1,13_=0.42 | | n.s. |  | | F_1,13_=0.06 | | n.s. | F_1,14_=6.70 | p<0.05 | | |  | F_1,14_=0.11 | n.s. | |  | | F_1,14_=0.00 | n.s. |
| **Motor cortex-Cerebellum** | F_1,13_=0.74 | n.s. |  | F_1,13_=0.00 | | n.s. |  | | F_1,13_=2.35 | | n.s. | F_1,14_=0.39 | n.s. | | |  | F_1,14_=0.99 | n.s. | |  | | F_1,14_=0.45 | n.s. |
| **Motor cortex-HPC** | F_1,13_=0.09 | n.s. |  | F_1,13_=11.99 | | p<0.01 |  | | F_1,13_=1.27 | | n.s. | F_1,14_=0.06 | n.s. | | |  | F_1,14_=0.06 | n.s. | |  | | F_1,14_=0.27 | n.s. |
| **Cerebellum-HPC** | F_1,13_=0.27 | n.s. |  | F_1,13_=1.33 | | n.s. |  | | F_1,13_=0.00 | | n.s. | F_1,14_=1.87 | n.s. | | |  | F_1,14_=0.14 | n.s. | |  | | F_1,14_=1.62 | n.s. |

**Table S3.** Two-way ANOVA (treatment (T) and genotype (G) as factors) for functional connectivity in male (♂) and female (♀) WT and GluN2CKO mice. Prefrontal cortex (PFC), hippocampus (HPC), caudate-putamen nuclei (CPu).
