# Supplementary figures and images for: Involvement of NMDA receptors containing the GluN2C subunit in the psychotomimetic and antidepressant-like effects of ketamine
Source: Transl Psychiatry. 2020 Dec 10;10:427. doi: 10.1038/s41398-020-01110-y (PMC7729946; doi:10.1038/s41398-020-01110-y)

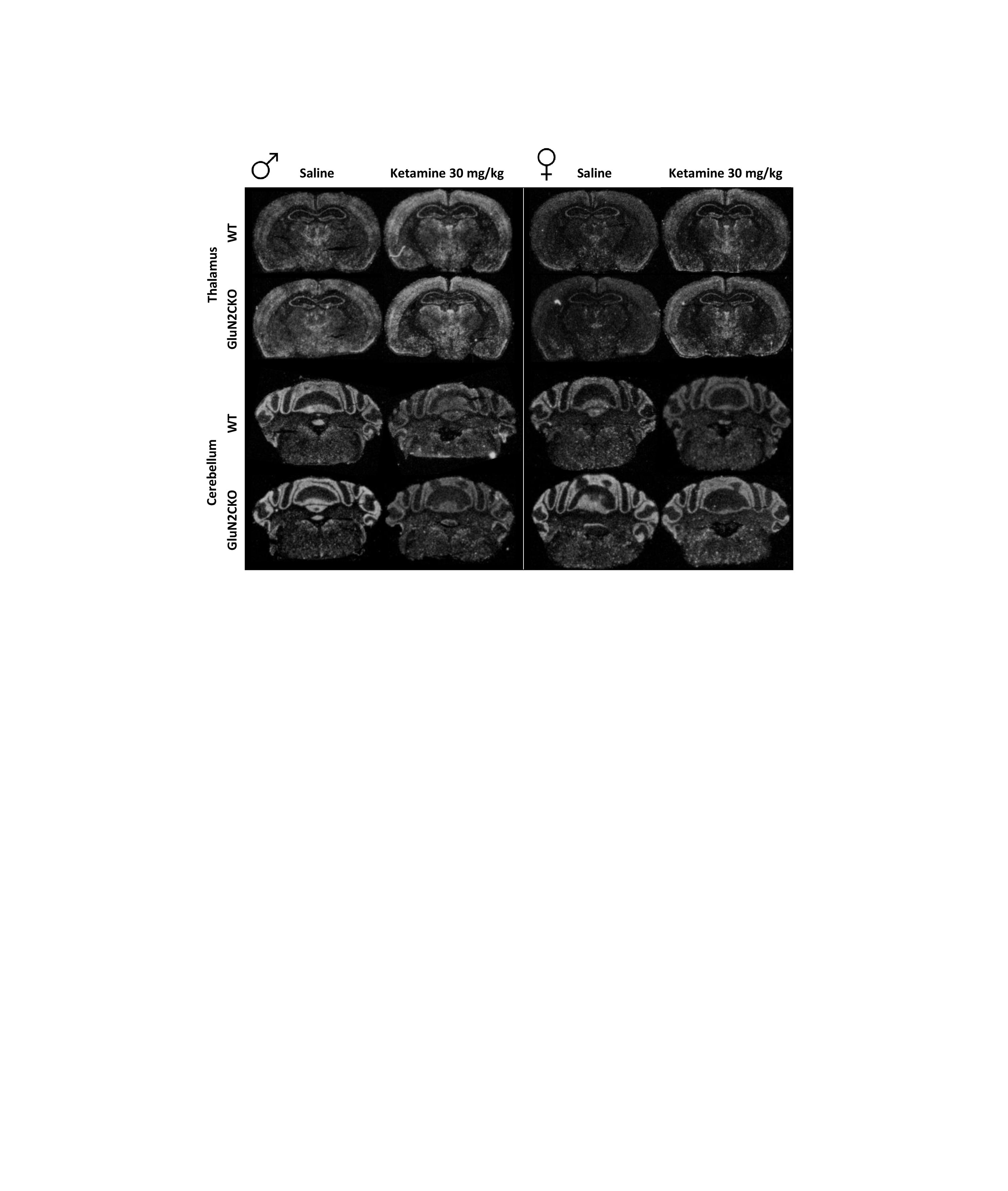

Supplement: Supplementary file 5 — Figure S1 [file 41398_2020_1110_MOESM5_ESM.jpg]

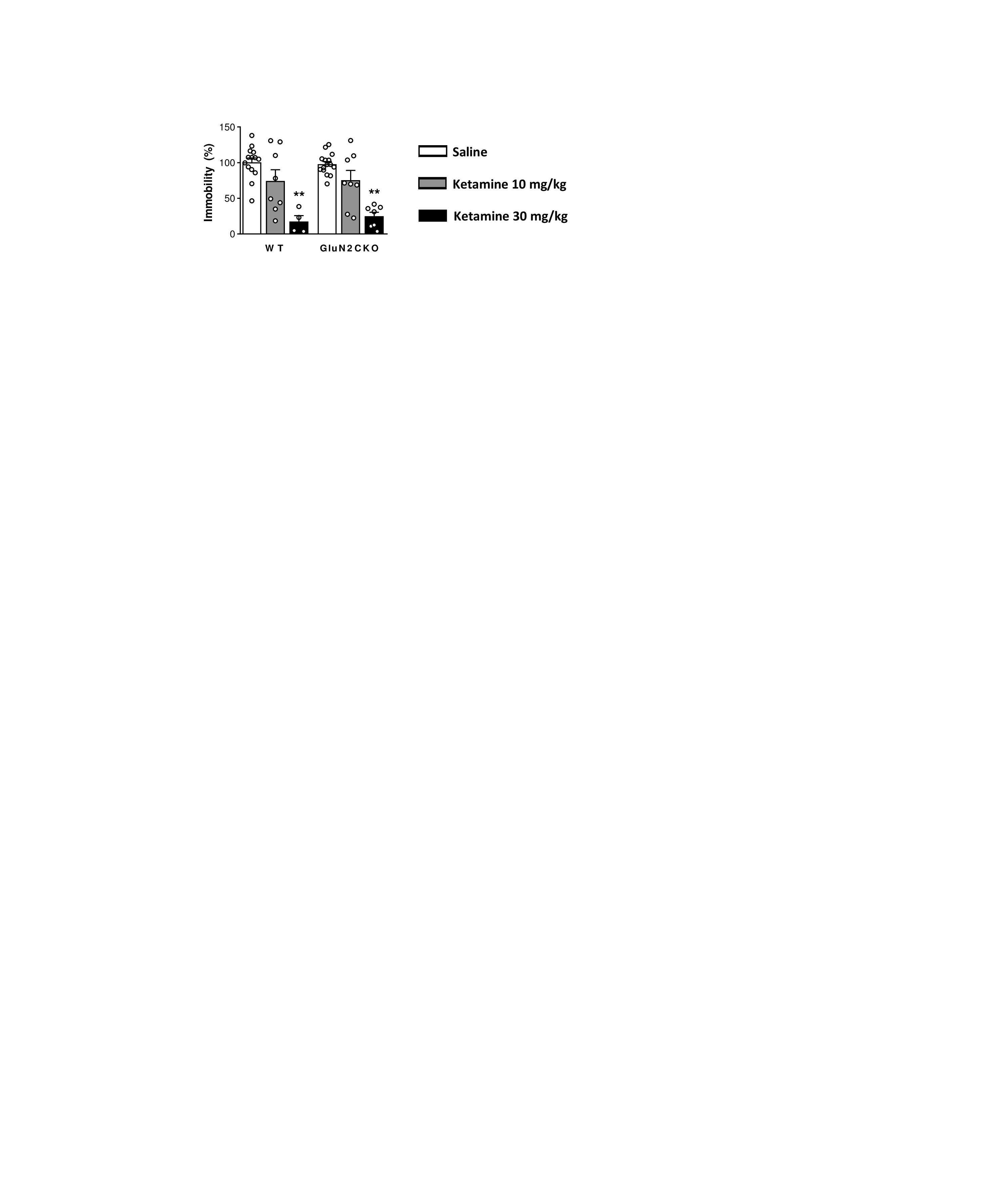

Supplement: Supplementary file 6 — Figure S2 [file 41398_2020_1110_MOESM6_ESM.jpg]
